# Stabilization of ruthenium on biochar-nickel magnetic nanoparticles as a heterogeneous, practical, selective, and reusable nanocatalyst for the Suzuki C–C coupling reaction in water†

**DOI:** 10.1039/d1ra09350a

**Published:** 2022-05-04

**Authors:** Parisa Moradi, Maryam Hajjami

**Affiliations:** Department of Chemistry, Faculty of Science, Ilam University P. O. Box 69315516 Ilam Iran; Department of Organic Chemistry, Faculty of Chemistry, Bu-Ali Sina University 6517838683 Hamedan Iran mhajjami@yahoo.com m.hajjami@basu.ac.ir

## Abstract

Waste recycling and the use of recyclable and available catalysts are important principles in green chemistry in science and industrial research. Therefore in this study, biochar nanoparticles were prepared from biomass pyrolysis. Then, they were magnetized with nickel nanoparticles to improve their recycling. Further, the magnetic biochar nanoparticles (biochar-Ni MNPs) were modified by dithizone ligand and then applied for the fabrication of a ruthenium catalyst (Ru-dithizone@biochar-Ni MNPs). This nanocatalyst was characterized by high-resolution transmission electron microscopy (HRTEM), scanning electron microscopy (SEM), energy-dispersive X-ray spectroscopy (EDS), wavelength dispersive X-ray spectroscopy (WDX), N_2_ adsorption–desorption isotherms, thermogravimetric analysis (TGA), X-ray diffraction (XRD), and vibrating sample magnetometry (VSM) techniques. The XRD studies of Ru in the nanocatalyst showed that the crystalline structure of ruthenium in the Ru-dithizone@biochar-Ni MNPs was *hcp*. Another principle of green chemistry is the use of safe and inexpensive solvents, the most suitable of which is water. Therefore, the catalytic activity of this catalyst was investigated as a practical, selective, and recyclable nanocatalyst in the Suzuki carbon–carbon coupling reaction in aqueous media. The VSM curve of this catalyst showed that it could be easily recovered using an external magnet, and recycled multiple times. Also, VSM analysis of the recovered catalyst indicated the good magnetic stability of this catalyst after repeated use.

## Abstract 2

Waste recycling and the use of recyclable and available catalysts are important principles in green chemistry in science and industrial research.

## Graphical abstract

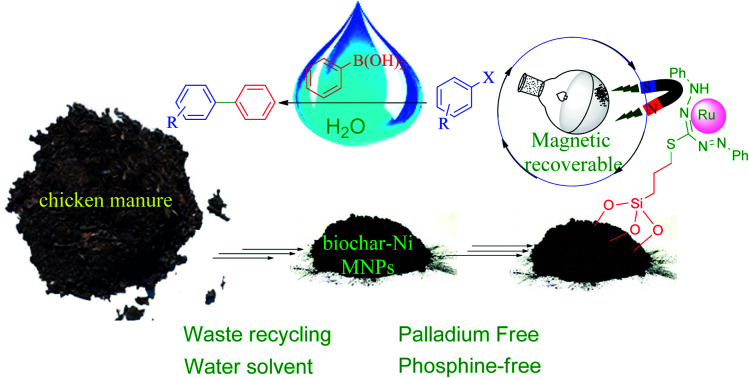

## 1 Introduction

Solvents, catalyst species and waste recycling are very important in green chemistry and industrial processes because waste species are very inexpensive and waste recycling prevents environmental pollution and material dissipation, and also as waste recycling offers an excellent manufacturing value-added proposition.^1–6^ Therefore, in this study, biochar nanoparticles were prepared from chicken manure, which introduces a method for waste recycling in the field of catalyst science.^5,6^

Another principle of green chemistry is to improve the stability and recycling of catalysts. Homogeneous catalysts tend to have better efficiency and selectivity than heterogeneous catalysts,^7–9^ but the reuse of homogeneous catalysts are often difficult, expensive, and time consuming.^9^ In this field, nanocatalysts have emerged because nanocatalysts have a very high surface area and act like homogeneous catalysts in terms of their catalytic activity.^10,11^ On the other hand, like heterogeneous catalysts, they can be recovered and reused.^9–11^ However, nanocatalysts cannot fully be recovered by inexpensive and conventional methods such as filtration due to their very small size. So, based on clean technology, magnetic nanoparticles (MNPs) have received special attention from scientists in the catalytic field because MNPs can be easily and completely recovered by an external magnet.^12^ Accordingly, in this study, we magnetized biochar nanoparticles using nickel MNPs. Then, we used the biochar MNPs (biochar-Ni) as a support to stabilize a ruthenium catalyst. The application of this catalyst was studied in a carbon–carbon coupling reaction in aqueous media. As known, carbon–carbon coupling reactions are usually performed in the presence of palladium catalysts containing phosphine ligands, which are expensive, unstable, and non-reusable.^13–17^ Therefore, the development of phosphine-free and palladium-free methods is significant due to their greater environmental compatibility.^18–21^ C–C bond formation was chosen here as it is a powerful tool in organic chemistry for the preparation of many natural products, biologically active compounds, hydrocarbons, and advanced materials.^22–24^

Another principle of green chemistry is the use of safe, available, and environmentally friendly solvents, such water, ethanol, PEG, and ionic liquids. Among these, water is the cheapest and safest solvent available.^25–32^ Further, non-polar organic compounds are not soluble in water, which makes it very easy to purify the final products in many reactions.^29–32^ Therefore, in this work, biochar nanoparticles were prepared and magnetized by nickel nanoparticles to facilitate recovery. Then, a ruthenium catalyst was fabricated based on the biochar MNPs. Finally, the application of this catalyst was studied in the carbon–carbon coupling reaction in aqueous media.

## 2 Experimental

### 2.1 Synthesis of magnetic nickel nanoparticles (Ni MNPs)

**Scheme 1:**
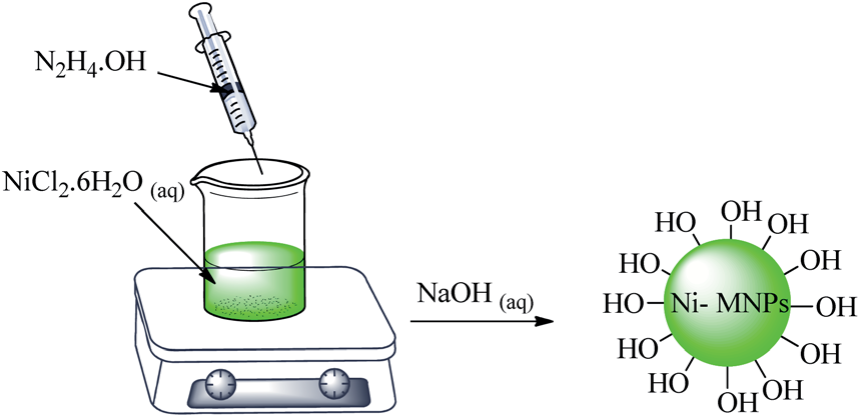
Synthesis of nickel magnetic nanoparticles (Ni MNPs).

A mixture of ethylene glycol (30 mL) and NiCl_2_·6H_2_O (0.5 g) was stirred and heated up to 60 °C. Then, 1.4 mL of hydrazine hydrate was added drop wise. Afterward, sodium hydroxide solution (1 M, 3.6 mL) was added and a black suspension was formed after 5 min. This suspension was stirred for 1 h at 60 °C. Finally, the magnetic nickel nanoparticles (Ni MNPs) were separated using an external magnet. The Ni MNPs were washed with deionized water several times and finally dried at room temperature (Scheme 1).

### 2.2 Synthesis of magnetic biochar nanoparticles using nickel magnetic nanoparticles (biochar-Ni MNPs)

Biochar nanoparticles were synthesized according to a previously reported procedure.^33^ Subsequently, the biochar nanoparticles (30 mg) were dispersed in deionized water (50 mL) for 20 min. Then, Ni MNPs (10 mg) were added to the mixture and dispersed again for another 20 min. Afterward, the mixture was stirred for 24 h at room temperature. Finally, the biochar-Ni MNPs were isolated by an external magnet. The obtained biochar-Ni MNPs were washed with deionized water and dried at 50 °C.

### 2.3 Stabilization of ruthenium on the biochar-Ni MNPs for the preparation of Ru-dithizone@biochar-Ni MNPs

**Scheme 2:**
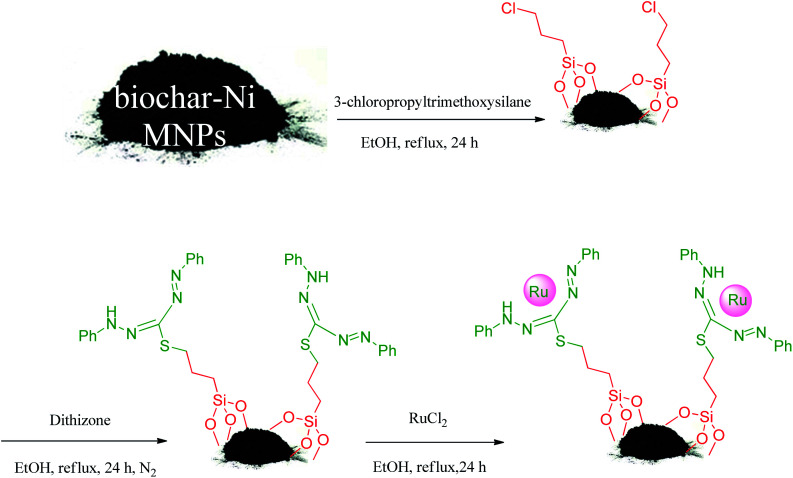
Preparation of Ru-dithizone@biochar-Ni MNPs.

First, the biochar-Ni MNPs (1 g) were dispersed in *n*-hexane (25 mL) for 30 min. Afterward, 1.5 mL of 3-chloropropyltrimethoxysilane was added to the mixture and stirred for 24 h under reflux conditions. The formed powder (*n*-pr-Cl@biochar-Ni MNPs) was obtained *via* an external magnet using ethanol washing followed by drying at 50 °C. Next, the *n*-pr-Cl@biochar-Ni MNPs (1 g) were dispersed in ethanol (25 mL) for 30 min. Then 3 mmol of dithizone was added to the reaction mixture and the mixture was refluxed under a N_2_ atmosphere for 24 h. The formed powder (dithizone@biochar-Ni MNPs) was isolated *via* an external magnet using ethanol washing followed by drying at 50 °C. Finally, the dithizone@biochar-Ni MNPs (1 g) were dispersed in ethanol (25 mL) for 20 min. Then, RuCl_3_ (0.3 g) was added and the mixture was refluxed under a N_2_ atmosphere for 24 h. The final product (Ru-dithizone@biochar-Ni MNPs) was washed by water and ethanol and obtained by magnetic decantation. The final catalyst was dried at 50 °C (Scheme 2).

### 2.4 General procedure for the synthesis of biphenyls

A mixture of Na_2_CO_3_ (3 mmol, 0.318 g), aryl halide (1 mmol), 1 mmol (0.122 g) of phenylboronic acid (PhB(OH)_2_), and Ru-dithizone@biochar-Ni MNPs (20 mg) was stirred in H_2_O at 80 °C. The end of the reaction was determined using TLC paper. After completion of the reaction, the reaction mixture was cooled down to room temperature. The catalyst was removed by an external magnet and washed using ethyl acetate. The products were extracted using H_2_O and ethyl acetate. The organic phase was dried by anhydrous Na_2_SO_4_. Then the organic solvents were evaporated and pure biphenyl derivatives were obtained.

### 2.5 Selected NMR data

#### 2.5.1 4-Nitro-1,1′-biphenyl

^1^H NMR (400 MHz, CDCl_3_): *δ*_H_ = 8.33–8.30 (d, *J* = 12 Hz, 2H), 7.77–7.74 (d, *J* = 12 Hz, 2H), 7.66–7.61 (m, 2H), 7.54–7.45 (m, 3H) ppm. IR (KBr) cm^−1^: 3076, 2932, 2838, 1967, 1930, 1596, 1576, 1514, 1479, 1449, 1404, 1346, 1296, 1286, 1159, 1116, 1105, 1079, 1006, 973, 926, 854, 774, 741, 700, 693. Raman: 3183, 3068, 2928, 1926, 1867, 1676, 1605, 1475, 1423, 1386, 1348, 1295, 1187, 1160, 1110, 1033, 999, 848, 749, 414.

#### 2.5.2 [1,1′-Biphenyl]-4-carbaldehyde

^1^H NMR (400 MHz, CDCl_3_): *δ*_H_ = 10.07 (s, 1H), 7.99–7.96 (d, *J* = 12 Hz, 2H), 7.78–7.76 (d, *J* = 8 Hz, 2H), 7.67–7.64 (m, 2H), 7.53–7.42 (m, 3H) ppm. IR (KBr) cm^−1^: 3364, 3062, 3031, 2834, 2741, 2543, 1933, 1891, 1811, 1700, 1603, 1581, 1562, 1516, 1485, 1450, 1412, 1384, 1318, 1306, 1287, 1214, 1184, 1168, 1109, 1077, 1038, 1006, 940, 920, 864, 837, 765, 730, 719, 697, 646, 628. Raman: 3195, 3064, 1701, 1610, 1569, 1512, 1412, 1289, 1214, 1176, 1110, 1039, 999, 830, 720, 638, 608, 478, 408.

#### 2.5.3 4-Chloro-1,1′-biphenyl

IR (KBr) cm^−1^: 3058, 2890, 2824, 1953, 1900, 1774, 1664, 1582, 1475, 1395, 1339, 1095, 1001, 909, 831, 756, 686, 542, 470. Raman: 3179, 3072, 1931, 1797, 1656, 1605, 1506, 1284, 1236, 1209, 1160, 1105, 1039, 999, 824, 760, 655, 608, 414, 323, 280.

## 3 Results and discussion

### 3.1 Catalyst characterization

**Fig. 1:**
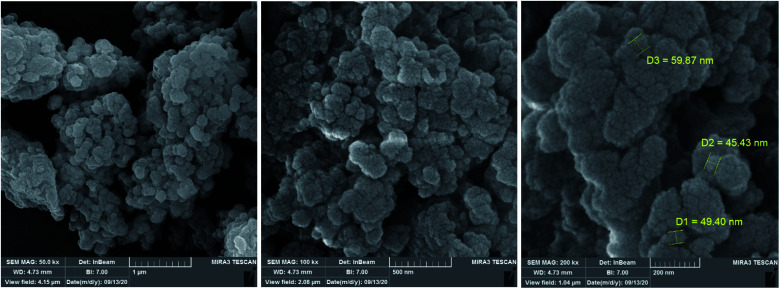
SEM images of Ru-dithizone@biochar-Ni MNPs.

After synthesis of the Ru-dithizone@biochar-Ni MNPs, this catalyst was characterized by various techniques, such as HRTEM, SEM, EDS, WDX, N_2_ adsorption–desorption isotherms, TGA, XRD, and VSM. SEM images of Ru-dithizone@biochar-Ni MNPs are shown in Fig. 1. Based on the SEM images, this catalyst had been synthesized in quasi-spherical shapes with a diameter of less than 60 nm. Agglomeration and adhesion is one of the characteristics of magnetic particles, therefore the agglomeration of nanoparticles observed in the SEM images indicated the magnetic nature of this catalyst.

**Fig. 2:**
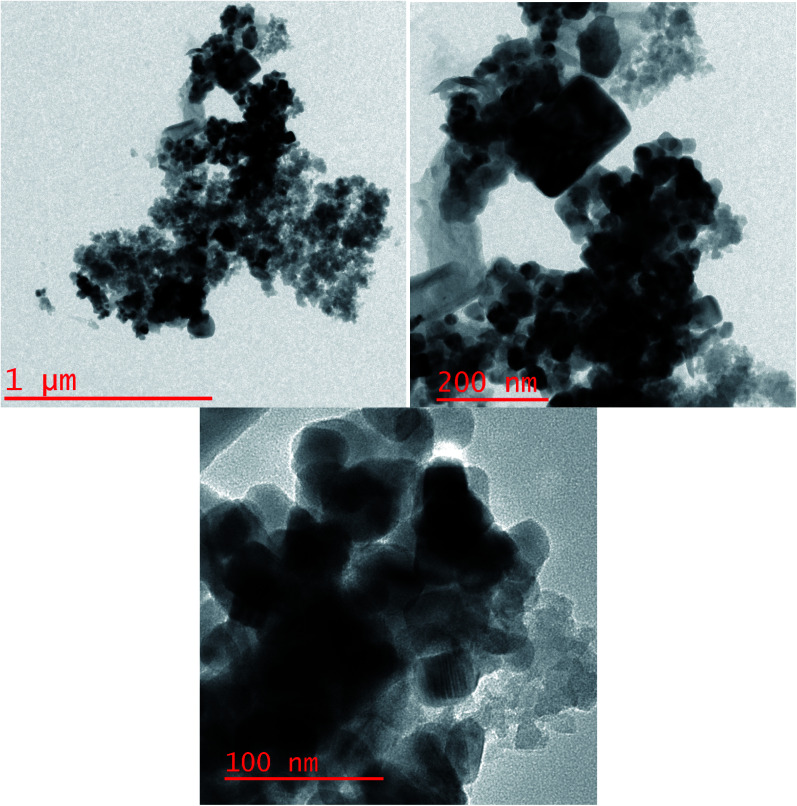
HRTEM images of Ru-dithizone@biochar-Ni MNPs.

Also, HRTEM images of Ru-dithizone@biochar-Ni MNPs are shown in Fig. 2, which confirmed the obtained results from the SEM analysis. The HRTEM images showed the quasi-spherical shapes of the particles with a diameter of less than 60 nm.

**Fig. 3:**
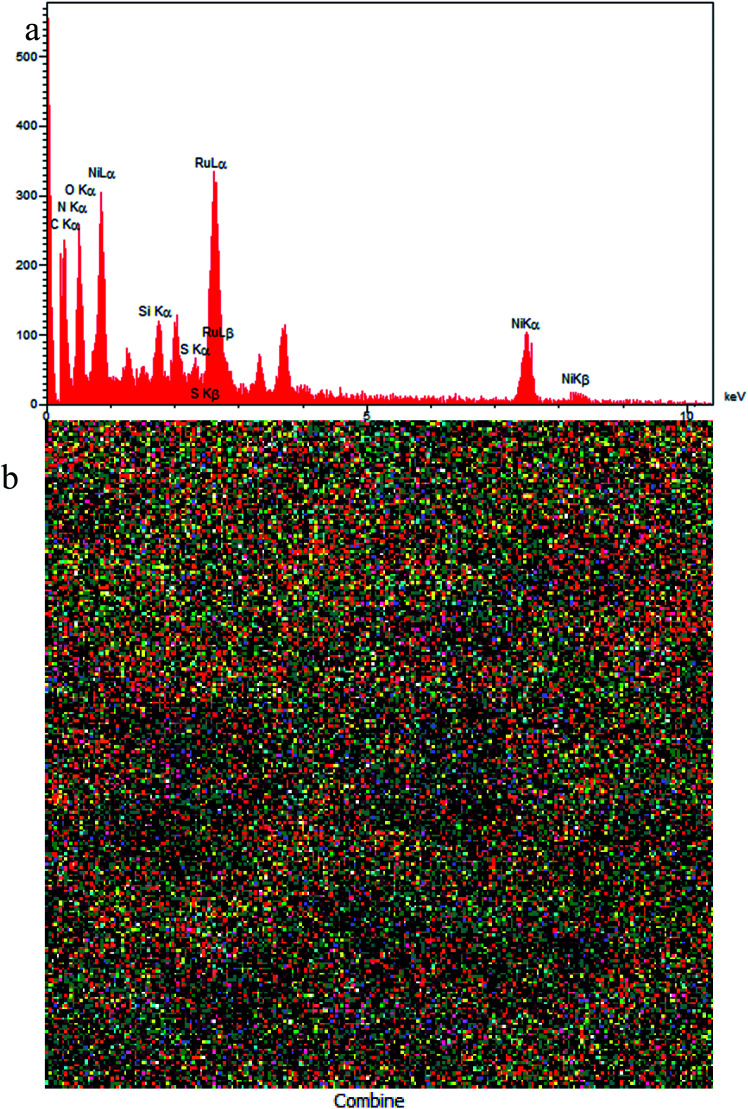
EDS spectrum (a) and elemental mapping (b) of Ru-dithizone@biochar-Ni MNPs.

The elemental composition of Ru-dithizone@biochar-Ni MNPs was qualitatively studied using WDX and EDS analysis (Fig. 3a). As expected, this catalyst was composed from C, O, Si, Ni, S, N, and Ru elements, which was in good agreement with the structure of Ru-dithizone@biochar-Ni MNPs in Scheme 2. Also, the obtained result from the EDS analysis were confirmed by the WDX analysis, which is shown in Fig. 3b. Based on the WDX analysis, all the elements were homogeneously distributed in the catalyst structure.

**Fig. 4:**
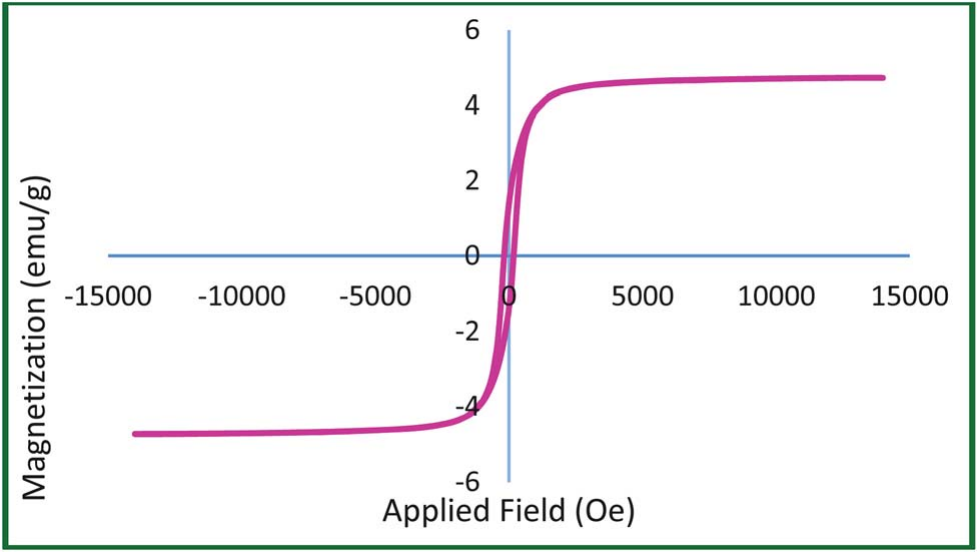
Magnetization curve for Ru-dithizone@biochar-Ni MNPs.

Room temperature VSM analysis of Ru-dithizone@biochar-Ni MNPs was performed to study the magnetic property of this catalyst (Fig. 4). As shown, this catalyst had a good magnetic property, with a magnetization value of about 4.72 emu g^−1^. As expected, the magnetization value of Ru-dithizone@biochar-Ni MNPs was lower than the magnetization value of Ni MNPs, which was reported to be about 45.71 emu g^−1^.^34^ This decrease in magnetic property was related to the insulation of the biochar and the grafting of organic layers on the surface of the biochar MNPs. However, the VSM curve of Ru-dithizone@biochar-Ni MNPs showed a good magnetic property of this catalyst, which confirmed its magnetic recyclability.

**Fig. 5:**
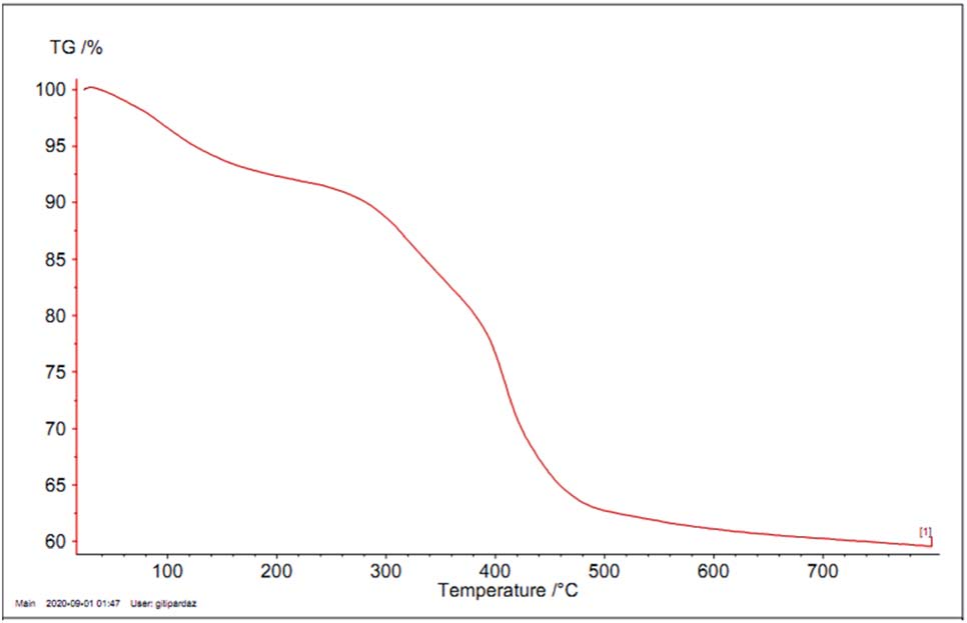
TGA diagram of Ru-dithizone@biochar-Ni MNPs.

Thermogravimetric analysis (TGA) of Ru-dithizone@biochar-Ni MNPs was performed and the result is shown in Fig. 5, which was performed over an air flow from 30–800 °C with a heating ramp of 10 °C min^−1^. The TGA diagram of Ru-dithizone@biochar-Ni MNPs indicated there were three mass losses from 30–800 °C. The first slight weight loss process was at low temperatures between 25–150 °C for this catalyst. This slight weight loss was attributed to the evaporation of the adsorbed solvents,^33,35^ which accounted for 7% of the weight. The second big weight loss process was observed at 200–500 °C, which involved a major decrease in mass (about 29%). This weight loss indicated the decomposition of the supported organic moieties.^36,37^ The third slight weight loss process occurred above 500 °C, which accounted for about 2% of the mass. This weight loss may be due to the continuing biochar pyrolysis.

**Fig. 6:**
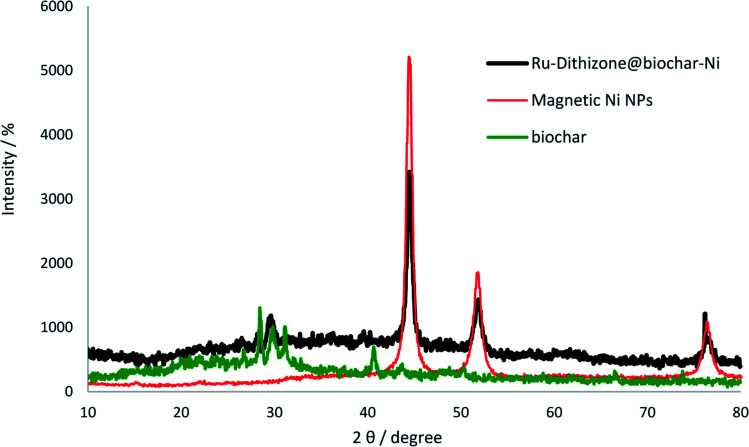
Normal XRD patterns of Ni MNPs, biochar NPs, and Ru-dithizone@biochar-Ni MNPs.

The structures of the Ni MNPs, biochar NPs, and Ru-dithizone@biochar-Ni MNPs were characterized by XRD (Fig. 6) using a PW1730 instrument from Philips and using CuKα (*λ* = 1.540598 Å) radiation at 40 kV and 30 mA with 2*θ* = 10–80°. The face-centered cubic structure (*fcc*) of Ni MNPs was characterized by three peaks of 2*θ* value at 44.5°, 51.5°, and 76.7° in the normal XRD pattern of Ni MNPs (Fig. 6, red line), which corresponded to the Miller indices (1 1 1), (2 0 0), and (2 2 0), respectively.^38–41^ The normal XRD pattern of the biochar NPs (Fig. 6, green line) showed a peak batch in the region of 2*θ* value at 30° ± 2, which corresponded to the Miller indices (0 0 2).^23,33^ Also, four peaks of 2*θ* were observed in the region of 40.8°, 44.0°, 48.2°, and 66.8°, corresponding to the biochar NPs.^23^ The 2*θ* peaks of Ni MNPs were not observed in the XRD spectrum of the biochar NPs, while the XRD pattern of Ru-dithizone@biochar-Ni MNPs (Fig. 6, black line) was included in both diffractions 2*θ* of biochar NPs and Ni MNPs, which confirmed the successful magnetization of the biochar NPs with the Ni MNPs. For example, the strong peaks of 2*θ* value at 44.5°, 52.5°, and 76.7° confirmed that the Ni MNPs had a good crystalline structure in Ru-dithizone@biochar-Ni MNPs;^42,43^ therefore the crystalline structured Ni MNPs did not any change after magnetization of the biochar NPs and their modification. Also, peaks at 2*θ* = 30°, 40.8°, 44.0°, 48.2°, and 66.8° in the XRD pattern of Ru-dithizone@biochar-Ni MNPs indicated the stability of the biochar nanoparticles after magnetization and modification. According to authentic reports, the crystalline structure of ruthenium can exist in two forms, *fcc* or *hcp*. The *fcc* crystalline structure of ruthenium corresponds to the 111, 200, 220, 311, and 222 reflections of the Miller indices, and the *hcp* crystalline structure of ruthenium corresponds to the 100, 002, 101, 102, 210, 103, 212, and 201 reflections of the Miller indices.^44^ In *fcc* and *hcp* crystalline structured ruthenium, the strong intensity corresponded to the (111, 2*θ* ∼ 40°) and (101, 2*θ* ∼ 44°) reflections, respectively. In the XRD pattern of Ru-dithizone@biochar-Ni MNPs, no peaks were observed in the area of 2*θ* ∼ 40° (111) for the *fcc* crystalline, while the peak in the area of 2*θ* ∼ 44° (101) for the *hcp* crystalline strongly overlapped with the peak of 2*θ* value at 44.5° from Ni MNPs. Also, two weak peaks were observed at 2*θ* = 38.8° (100) and 77.5° (103) in the XRD pattern of Ru-dithizone@biochar-Ni MNPs, which were related to the *hcp* crystalline structure of ruthenium, while the *fcc* crystalline structure did not have these peaks. Also, the *fcc* crystal structure of ruthenium typically has a peak in the region of 2*θ* ∼ 40° (200), which was not seen in the XRD pattern of Ru-dithizone@biochar-Ni MNPs. Therefore, it is possible that the crystalline structure of ruthenium in the Ru-dithizone@biochar-Ni MNPs was *hcp*.

**Fig. 7:**
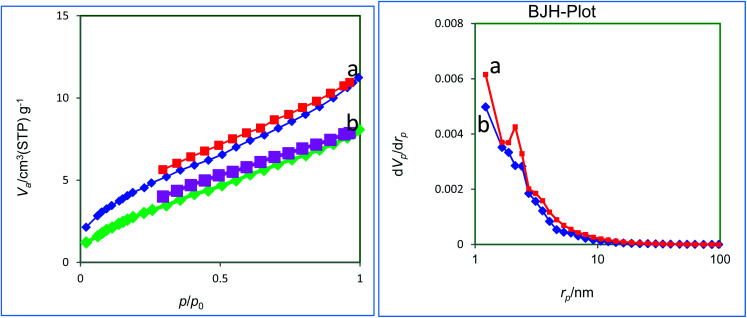
N_2_ adsorption–desorption isotherm and BJH-plot of biochar-Ni MNPs (a) and Ru-dithizone@biochar-Ni MNPs (b).

The N_2_ adsorption–desorption isotherms and BJH-plot of the biochar-Ni MNPs and Ru-dithizone@biochar-Ni MNPs are shown in Fig. 7. Based on the Brunauer–Emmett–Teller (BET) method, the BET surface area (199–557 m^2^ g^−1^),^33^ pore volumes (0.03–0.22 cm^3^ g^−1^),^33^ and pore diameters [2.4–3.0 nm]^33^ of the biochar were higher than for the biochar-Ni MNPs and Ru-dithizone@biochar-Ni MNPs. Besides, the BET surface area of biochar-Ni MNPs was 16.71 m^2^ g^−1^, which was higher than the BET surface area of Ru-dithizone@biochar-Ni MNPs (11.77 m^2^ g^−1^), which was due to the grafting of organic layers and Ru-complex on the surface of biochar nanoparticles. The total pore volumes of biochar-Ni MNPs and Ru-dithizone@biochar-Ni MNPs were 0.01 cm^3^ g^−1^, which indicated the organic layers and Ru-complex were fabricated on the surface of the biochar nanoparticles and not into its pores.

### 3.2 Catalytic application of Ru-dithizone@biochar-Ni MNPs

**Scheme 3:**
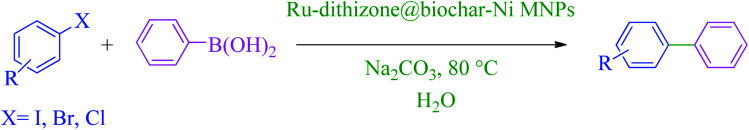
C–C coupling reaction of PhB(OH)_2_ with aryl halides in the presence of Ru-dithizone@biochar-Ni MNPs.

The catalytic application of Ru-dithizone@biochar-Ni MNPs was investigated in the carbon–carbon coupling reaction to synthesize biphenyl derivatives (Scheme 3). To optimize the reaction conditions, the coupling of phenylboronic acid (PhB(OH)_2_) with iodobenzene was studied in different conditions (Table 1). First, the effect of protic (Table 1, entries 2 and 3) and aprotic (Table 1, entries 4 and 5) polar solvents were examined. Among these, the best result was observed in water (Table 1, entry 7). Next, the model reaction was studied in the presence of different amount of Ru-dithizone@biochar-Ni MNPs in water (Table 1, entries 3, 6, and 7). As shown, this reaction did not progress in the absence of catalyst, and 20 mg of the catalyst was required to complete the reaction in a suitable time. Reducing the amount of the catalyst led to a longer reaction time or reduction in the yield of the product (Table 1, entry 3). Afterward, the influence of several bases (Na_2_CO_3_, NaOH, NaHCO_3_, Et_3_N, and K_2_CO_3_) was studied in the model reaction (Table 1, entries 7–11), in which Na_2_CO_3_ showed the best results at 80 °C (Table 1, entry 3). As shown in entry 12 of Table 1, a 20 °C decrease in reaction temperature (from 80 °C to 60 °C) led to a sharp decrease in the yield of the product (Table 1, entry 12). Based on the aforementioned studies, the optimal conditions for the C–C coupling reaction were obtained in the presence of 20 mg of Ru-dithizone@biochar-Ni MNPs at 80 °C in water using Na_2_CO_3_ as a base (Table 1, entry 7).

**Table 1:** Investigation of the optimal conditions for the C–C coupling reaction of PhB(OH)_2_ with iodobenzene in the presence of Ru-dithizone@biochar-Ni MNPs

Entry	Amount of catalyst (mg)	Solvent	Base	Temperature (°C)	Time (min)	Yield (%)
1	—	PEG	Na_2_CO_3_	80	420	N.R.
2	10	PEG	Na_2_CO_3_	80	260	85
3	10	H_2_O	Na_2_CO_3_	80	180	91
4	10	DMSO	Na_2_CO_3_	80	375	90
5	10	DMF	Na_2_CO_3_	80	305	87
6	15	H_2_O	Na_2_CO_3_	80	140	93
7	20	H_2_O	Na_2_CO_3_	80	90	96
8	20	H_2_O	NaHCO3	80	90	64
9	20	H_2_O	NaOH	80	90	46
10	20	H_2_O	Et_3_N	80	90	81
11	20	H_2_O	K_2_CO_3_	80	90	73
12	20	H_2_O	Na_2_CO_3_	60	90	38

After optimization of the reaction conditions, the scope of the catalytic activity of Ru-dithizone@biochar-Ni MNPs was extended to the cross C–C coupling of PhB(OH)_2_ with other aryl halides (Table 2). Therefore, several aryl chlorides, aryl bromides, and aryl iodides were investigated in the coupling reaction with PhB(OH)_2_ in the presence of Ru-dithizone@biochar-Ni MNPs. As shown in Table 2, the aryl iodides were coupled with PhB(OH)_2_ at the highest rate, while the aryl chlorides reacted with PhB(OH)_2_ at the lowest rate in the presence of Ru-dithizone@biochar-Ni MNPs. For example, the coupling of iodobenzene, bromobenzene, and chlorobenzene were observed in 90, 110, and 125 min, respectively. Therefore, the coupling rate of the aryl halides with PhB(OH)_2_ in the presence of this catalyst followed the order: aryl chlorides < aryl bromides < aryl iodides. As expected, 2′-bromoacetophenone was coupled with PhB(OH)_2_ in a long reaction time due to its high steric hindrance (Table 2, entry 12). The influence of the electronic nature of the aromatic ring on the aryl halides was also investigated. For this issue, various electron-donating or electron-withdrawing functional groups on the aromatic ring of the aryl halides were investigated in the C–C coupling reaction with PhB(OH)_2_ in the presence of Ru-dithizone@biochar-Ni MNPs. The highest reaction rates were obtained for the aryl halides having an electron-withdrawing functional group rather than electron-donating functional group. For example, PhB(OH)_2_ was coupled with 4-nitrobromobenzen and 4-hydroxybromobenzen after 40 and 95 min, respectively. Finally, bicyclic aryl halide having two aromatic rings, such as 1-bromonaphthalene, was investigated in the coupling with PhB(OH)_2_ in the presence of Ru-dithizone@biochar-Ni MNPs (Table 2, entry 13).

**Table 2:** Catalytic C–C coupling reaction of aryl halides with PhB(OH)_2_ in the presence of Ru-dithizone@biochar-Ni MNPs

Entry	Aryl halide	Product	Time (min)	Yield (%)
1	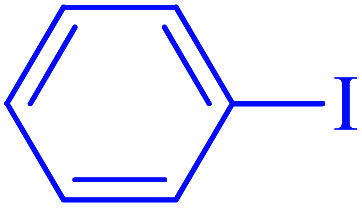	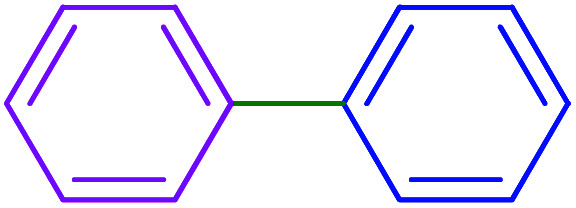	90	96
2	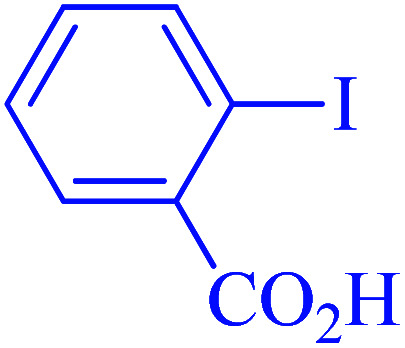	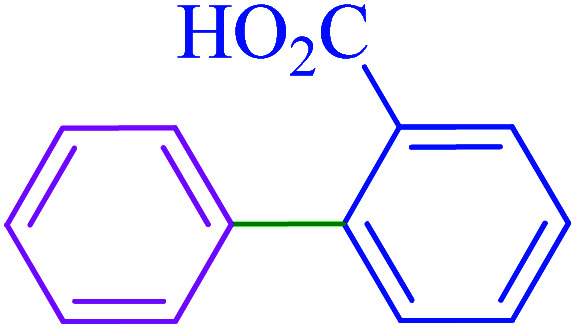	105	87
3	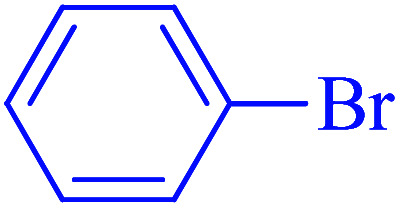	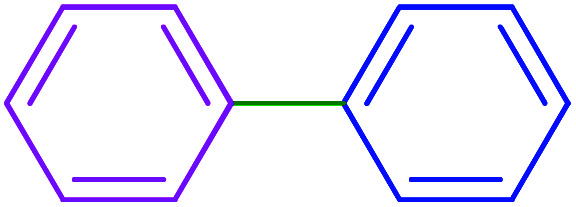	110	92
4	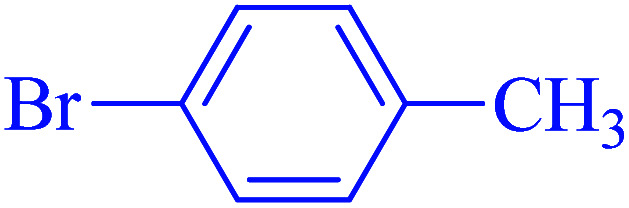	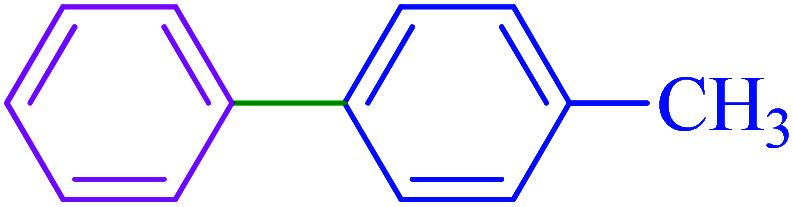	50	95
5	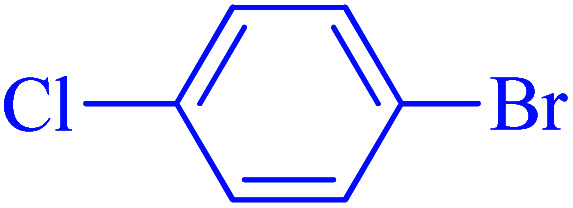	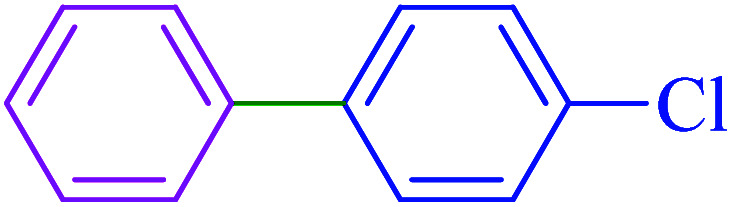	60	90
6	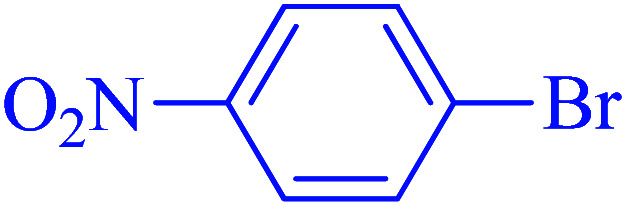	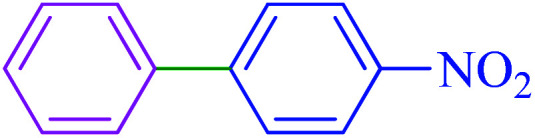	40	97
7	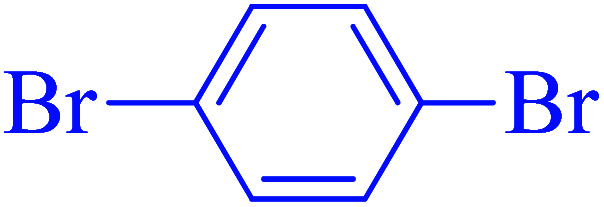	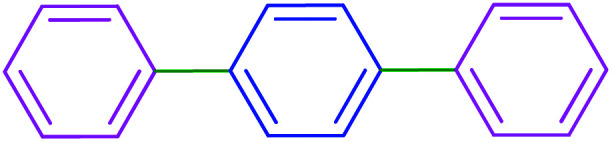	65	85
8	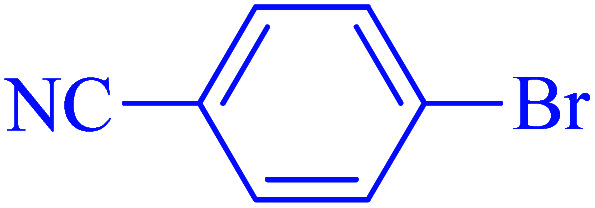	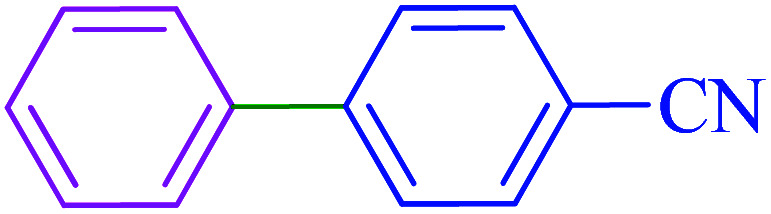	180	93
9	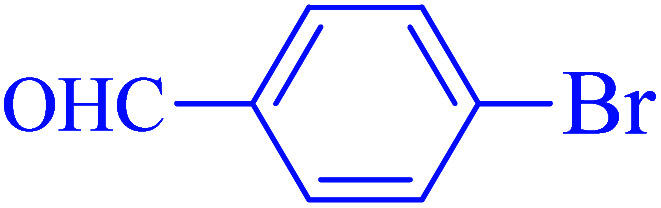	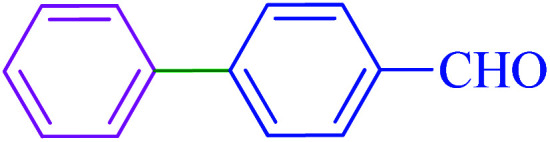	30 h	87
10	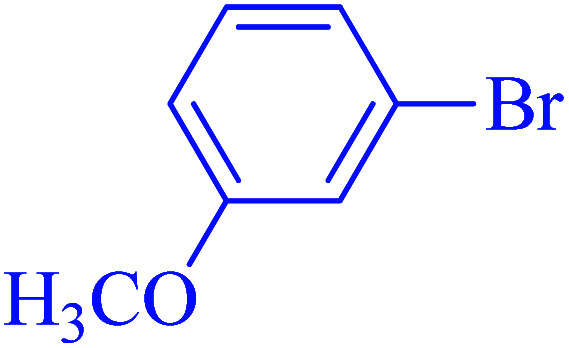	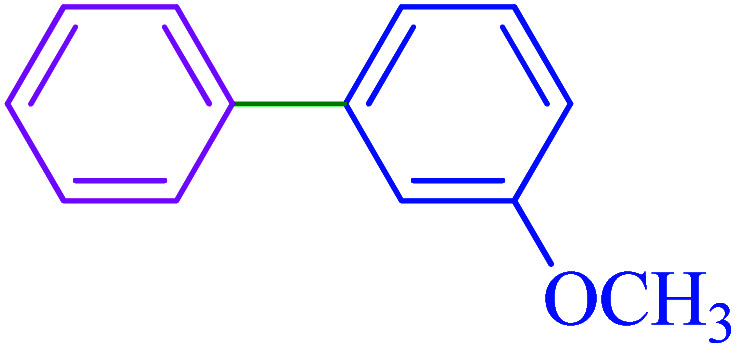	150	92
11	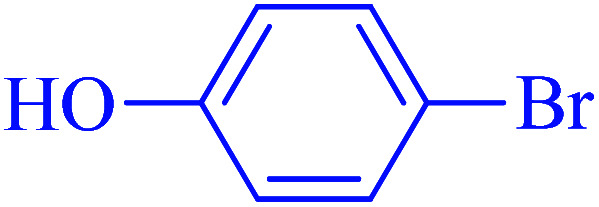	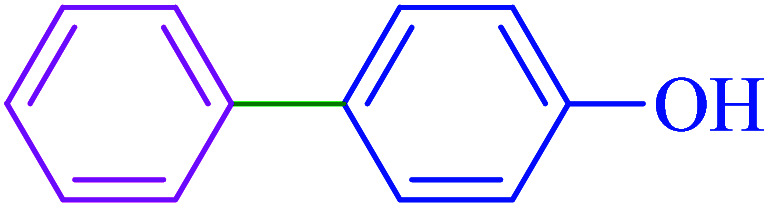	95	94
12	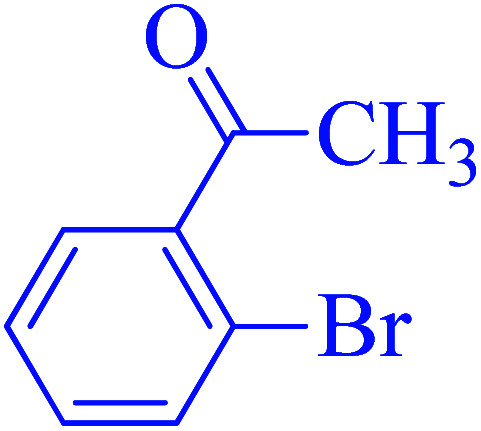	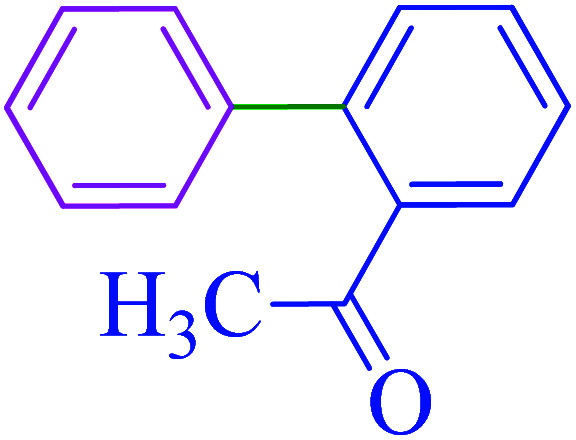	48 h	85
13	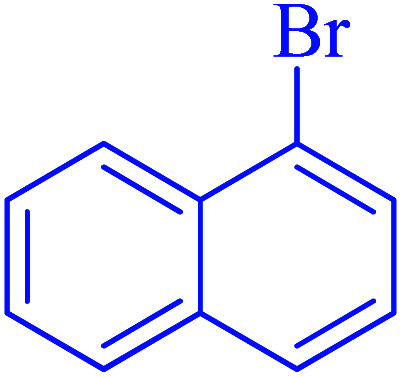	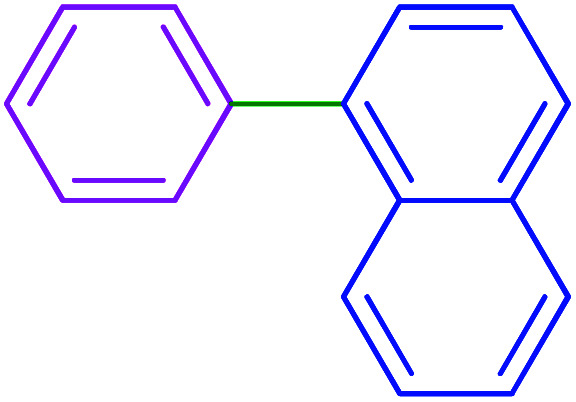	135	91
14	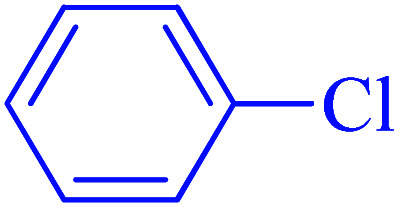	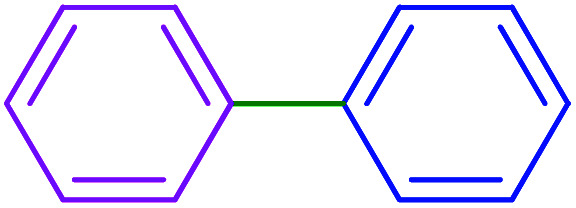	125	89
15	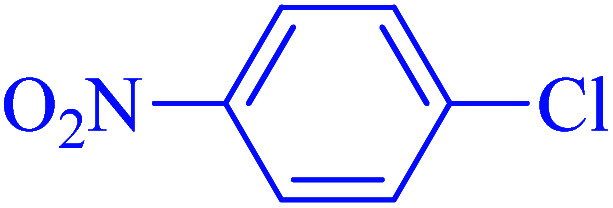	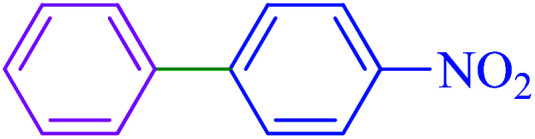	75	94
16	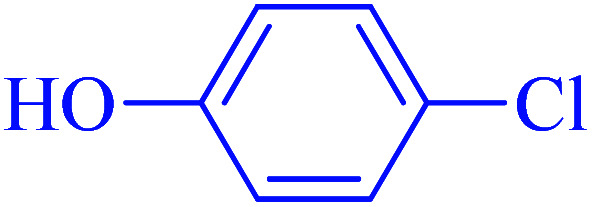	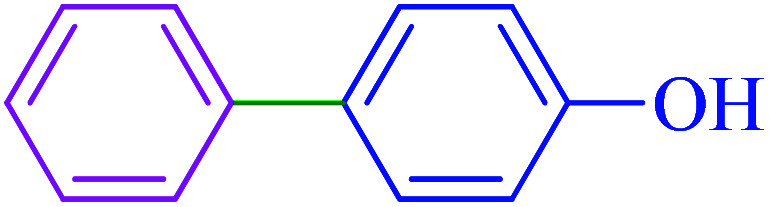	390	90
17	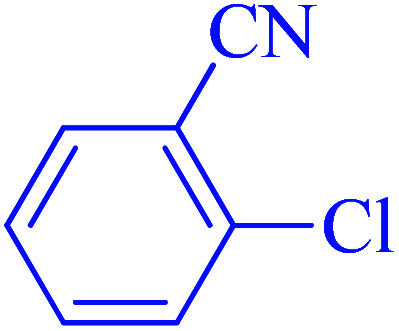	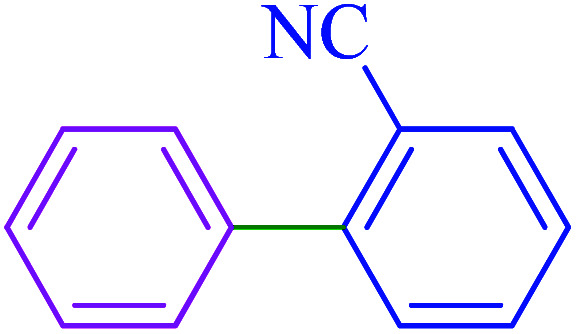	270	92

**Scheme 4:**
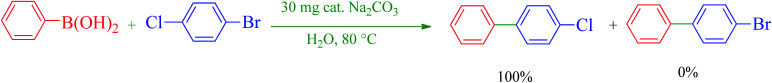
Selectivity in C–C coupling reactions in the presence of Ru-dithizone@biochar-Ni MNPs.

The selectivity of Ru-dithizone@biochar-Ni MNPs was studied in the coupling of PhB(OH)_2_ with 1-bromo-4-chorobenzene (Table 2, entry 5). As mentioned, the coupling speed of aryl bromides was greater than aryl chlorides. Therefore, in the coupling reaction of 1-bromo-4-chorobenzene with PhB(OH)_2_, only the coupling reaction was performed selectively from the aryl bromide side (Scheme 4). The FT-IR spectrum of 1-bromo-4-chlorobenzene was compared with that of 4-chloro-1,1′-biphenyl product. The C–Cl and C–Br stretching vibrations of 1-bromo-4-chlorobenzene appeared around 1069 cm^−1^, which overlapped together. However this vibrational peak for the 4-chloro-1,1′-biphenyl product appeared in the region around 1095 cm^−1^, which was related to the C–Cl bond alone (ESI, Fig. S3†). The FT-IR spectrum of the product corresponded exactly with the reported sources.^45,46^

**Table 3:** Comparison of Ru-dithizone@biochar-Ni MNPs in the C–C coupling reaction of iodobenzene or chlorobenzene with PhB(OH)_2_ with previously reported catalysts

Entry	Catalyst (mol%)	Aryl halide	Condition	Time (h)	Yield (%)	Ref.
1	Pd-imi-CC@MCM-41/Fe_3_O_4_	Chlorobenzene	Na_2_CO_3_, PEG, 80 °C	24 h	89	22
2	Pd(0)TBA@biochar	Chlorobenzene	Na_2_CO_3_, PEG, 80 °C	25 h	89	23
3	Pd-isatin-boehmite	Chlorobenzene	K_2_CO_3_, PEG, 80 °C	250	87	47
4	Pd(eao)_2_	Chlorobenzene	NaHCO_3_, PEG400/H_2_O, 80 °C	360	34	53
5	Pd@COF-QA	Chlorobenzene	TEA, H_2_O, 50 °C	360	99	54
6	Pd NPs@Fe_3_O_4_-lignin	Chlorobenzene	K_2_CO_3_, EtOH : H_2_O, 90 °C	270	81	55
7	Cross-linked poly (ITC-HPTPy)-Pd	Chlorobenzene	K_2_CO_3_, EtOH : H_2_O, 80 °C	600	96	56
8	HMS–CPTMS–Cy–Pd	Chlorobenzene	K_2_CO_3_, PEG, 100 °C	300	84	57
9	Pd(dba)_2_	Chlorobenzene	Click triazole, NaOBu^*t*^, toluene, 100 °C	18 h	80	58
10	Pd/Au NPs	Iodobenzene	EtOH/H_2_O, K_2_CO_3_, 80 °C	24 h	88	59
11	Cu-MPAMP@Fe_3_O_4_	Iodobenzene	Na_2_CO_3_, PEG, 80 °C	100	97	60
12	Copper powder	Iodobenzene	K_2_CO_3_, PEG, 110 °C	12 h	99	61
13	Pd NP	Iodobenzene	H_2_O, KOH, 100 °C	12 h	95	62
14	Pd-imi@MCM-41/Fe_3_O_4_	Chlorobenzene	Na_2_CO_3_, PEG, 80 °C	24 h	95	63
15	Cu–C	Iodobenzene	H_2_O, K_2_CO_3_, 50 °C	3.3 h	96	64
16	Cu–C	Chlorobenzene	H_2_O, K_2_CO_3_, 50 °C	240	15	64
17	Ru-dithizone@biochar-Ni	Chlorobenzene	Na_2_CO_3_, H_2_O, 80 °C	125	89	This work
18	Ru-dithizone@biochar-Ni	Iodobenzene	Na_2_CO_3_, H_2_O, 80 °C	90	96	This work

**Scheme 5:**
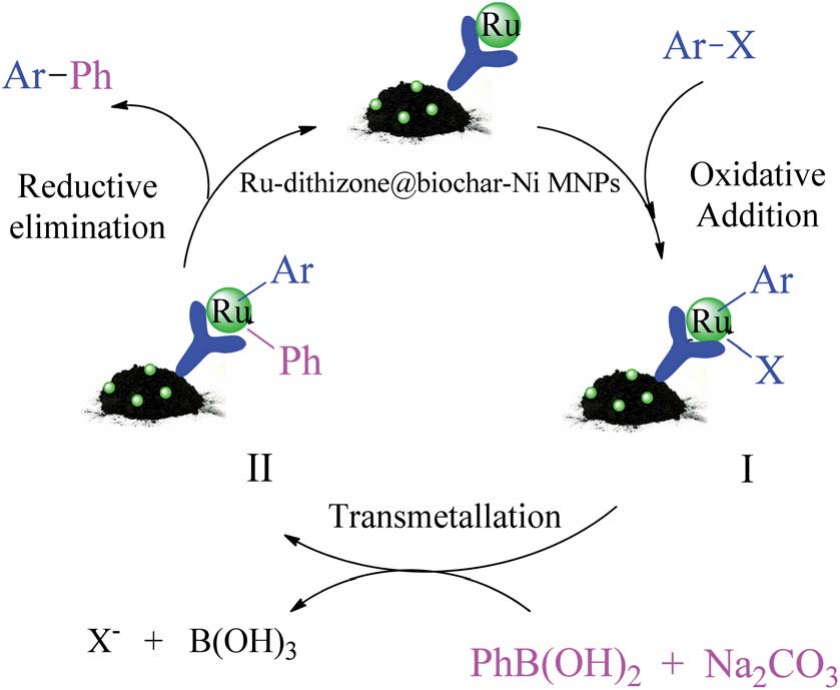
Suggested catalytic cycle for the Suzuki reaction in the presence of Ru-dithizone@biochar-Ni MNPs.

The catalytic cycle for the C–C coupling reaction in the presence of Ru-dithizone@biochar-Ni MNPs is suggested in Scheme 5.^47–52^ Because the dithizone ligand is chelated with ruthenium on one side, it creates a complex with little hindrance for catalytic application. Therefore, the metal catalyst center is easily accessible to the reactants. The suggested mechanism for the formation of biphenyl compounds is shown in Scheme 5. Biphenyl derivatives were synthesized in the presence of this catalyst in three steps (oxidative addition, transmetallation and reduction elimination) that form a catalytic cycle. In the first step, which is called oxidative addition, the oxidation number of the catalyst metal species increases and intermediate I is formed. In the second step, which called transmetallation, intermediate I is converted to intermediate II under the influence of the base and PhB(OH)_2_. Finally, the biphenyl derivatives are formed in the third step, which is called reduction elimination, and the catalyst is regenerated to continue the catalytic cycle. Based on this mechanism, polar solvents provided better conditions for the Suzuki reaction in the presence of a metal catalyst, which was consistent with the results in Table 1, and is because, polar solvents have the ability to solve a base and also to perform the solvation of the polar intermediates.

**Fig. 8:**
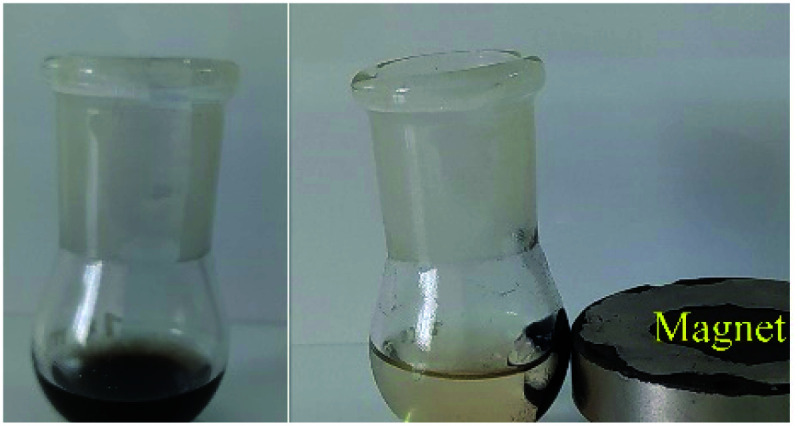
Reaction mixture in the absence (left) and presence (right) of an external magnet.

**Fig. 9:**
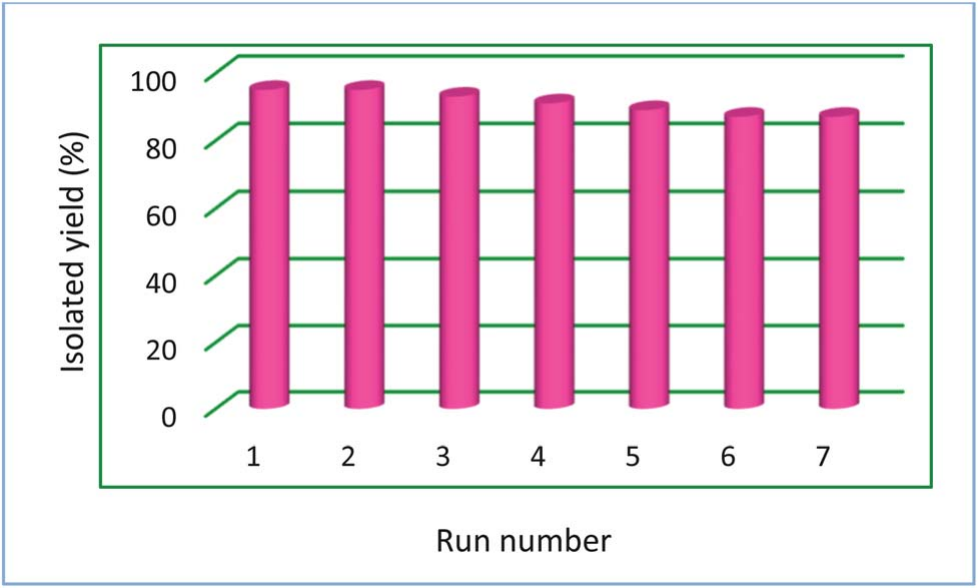
Recyclability of Ru-dithizone@biochar-Ni MNPs in the coupling of 4-bromotoluene with PhB(OH)_2_.

Due to the heterogeneous and magnetic nature of Ru-dithizone@biochar-Ni MNPs, they can be recovered easily with the assistance of an external magnet (Fig. 8) and reused for several times. As shown in Fig. 8, Ru-dithizone@biochar-Ni MNPs could be completely isolated using an external magnet for use again in the next runs. Therefore, the recoverability of this catalyst was examined in the synthesis of 4-methyl-1,1′-biphenyl by the cross-coupling reaction of 4-bromotoluene with PhB(OH)_2_. In this issue, this reaction was started under the optimized conditions and at the end of reaction, the catalyst was recovered using an external magnet. Then, the recovered catalyst was washed with ethyl acetate and was evaluated in the next run. This catalyst recovery cycle was repeated for 7 consecutive times, in which each time the products were obtained with high yield (Fig. 9). As shown in Fig. 9, the Ru-dithizone@biochar-Ni MNPs catalyst could be reused at least 7 times consecutively in C–C coupling reactions.

**Fig. 10:**
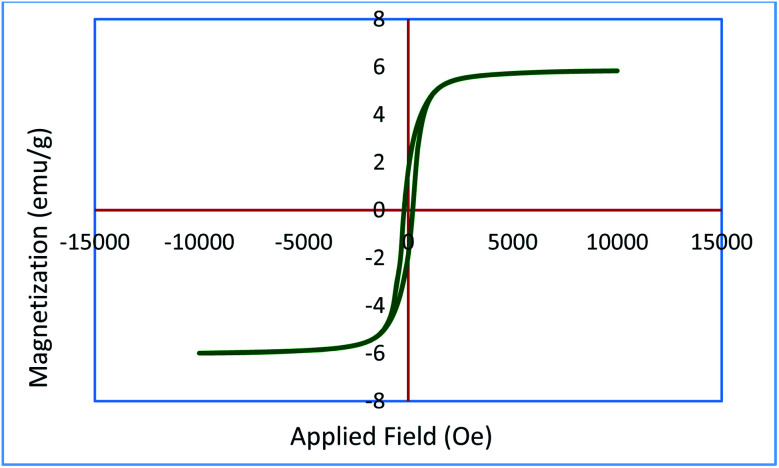
Magnetization curve for the reused Ru-dithizone@biochar-Ni MNPs.

To show the magnetic stability of Ru-dithizone@biochar-Ni MNPs after repeated use, the magnetic property of the recovered catalyst was characterized by VSM analysis. The magnetic value of the recovered catalyst was compared to the magnetic value of fresh catalyst (Fig. 10). As shown the magnetic value of the recovered catalyst was 5.83 emu g^−1^, which indicated a good agreement with the fresh catalyst in Fig. 4.

The efficiency and advantages of Ru-dithizone@biochar-Ni MNPs were compared with previous catalysts (Table 3) in the coupling of chlorobenzene or iodobenzene with PhB(OH)_2_. Most of the catalysts so far reported are made from chemical starting materials that are not economically viable or environmentally friendly. Whereas biochar is made from chicken manure, which is a great process for waste recycling. Therefore, it is both economically viable and environmentally friendly. Further, the C–C coupling reaction was carried out in aqueous media in the presence of Ru-dithizone@biochar-Ni MNPs; meanwhile toxic, expensive, and organic solvents were used in other works. Therefore, the Suzuki reaction in the presence of Ru-dithizone@biochar-Ni MNPs is completely consistent with the principles of green chemistry. Besides, the biphenyls were synthesized in the presence of Ru-dithizone@biochar-Ni MNPs in a shorter time with a higher yield than for the other catalysts. Also, in some cases, non-recyclable homogeneous catalysts have been used; whereas Ru-dithizone@biochar-Ni MNPs can be recovered using an external magnet, which can be reused over and over again.

## 4 Conclusions

In summary, according to principles of green chemistry and waste recycling, biochar nanoparticles were synthesized from the pyrolysis of chicken manure and then magnetized by nickel nanoparticles to improve their recycling. Then, the magnetic biochar nanoparticles were modified by 3-chloropropyltrimethoxysilane and dithizone ligand, respectively. Afterward, a Ru-complex was fabricated on the surface of the magnetic biochar nanoparticle nanoparticles (biochar-Ni MNPs) and evidenced using HRTEM, SEM, EDS, WDX, N_2_ adsorption–desorption isotherms, TGA, XRD, and VSM techniques. Finally, the catalytic activity of this catalyst was investigated as a practical, selective, and recyclable nanocatalyst in the carbon–carbon coupling reaction in water as a green solvent. It is worth noting, ruthenium has rarely been applied as a catalyst for the Suzuki coupling reaction. Interestingly this catalyst demonstrated a good catalytic practicality, selectivity, and reusability in the C–C coupling for a wide range of aryl chlorides, aryl bromides, and aryl iodides having an electron-donating or electron-withdrawing functional groups on *ortho*, *meta*, or *para* position of the aromatic ring. This catalyst could recovered and reused for 7 times at least without significant metal leaching or demagnetization.

## Conflicts of interest

The authors declare that they have no known competing financial interests or personal relationships that could have appeared to influence the work reported in this paper.

## Supplementary Material
